# Mitochondria-Targeted Antioxidants SkQ1 and MitoTEMPO Failed to Exert a Long-Term Beneficial Effect in Murine Polymicrobial Sepsis

**DOI:** 10.1155/2017/6412682

**Published:** 2017-09-19

**Authors:** Pia Rademann, Adelheid Weidinger, Susanne Drechsler, Andras Meszaros, Johannes Zipperle, Mohammad Jafarmadar, Sergiu Dumitrescu, Ara Hacobian, Luisa Ungelenk, Franziska Röstel, Jozsef Kaszaki, Andrea Szabo, Vladimir P. Skulachev, Michael Bauer, Soheyl Bahrami, Sebastian Weis, Andrey V. Kozlov, Marcin F. Osuchowski

**Affiliations:** ^1^Ludwig Boltzmann Institute for Experimental and Clinical Traumatology in the Trauma Research Center of AUVA, Vienna, Austria; ^2^Institute of Surgical Research, Faculty of Medicine, University of Szeged, Szeged, Hungary; ^3^Department of Anesthesiology and Intensive Care Medicine, Jena University Hospital, Jena, Germany; ^4^A.N. Belozersky Institute of Physico-Chemical Biology, M.V. Lomonosov Moscow State University, Leninskie Gory, Moscow 119992, Russia; ^5^Center for Sepsis Control and Care, Jena University Hospital, Jena, Germany

## Abstract

Mitochondrial-derived reactive oxygen species have been deemed an important contributor in sepsis pathogenesis. We investigated whether two mitochondria-targeted antioxidants (mtAOX; SkQ1 and MitoTEMPO) improved long-term outcome, lessened inflammation, and improved organ homeostasis in polymicrobial murine sepsis. 3-month-old female CD-1 mice (*n* = 90) underwent cecal ligation and puncture (CLP) and received SkQ1 (5 nmol/kg), MitoTEMPO (50 nmol/kg), or vehicle 5 times post-CLP. Separately, 52 SkQ1-treated CLP mice were sacrificed at 24 h and 48 h for additional endpoints. Neither MitoTEMPO nor SkQ1 exerted any protracted survival benefit. Conversely, SkQ1 exacerbated 28-day mortality by 29%. CLP induced release of 10 circulating cytokines, increased urea, ALT, and LDH, and decreased glucose but irrespectively of treatment. Similar occurred for CLP-induced lymphopenia/neutrophilia and the NO blood release. At 48 h post-CLP, dying mice had approximately 100-fold more CFUs in the spleen than survivors, but this was not SkQ1 related. At 48 h, macrophage and granulocyte counts increased in the peritoneal lavage but irrespectively of SkQ1. Similarly, hepatic mitophagy was not altered by SkQ1 at 24 h. The absence of survival benefit of mtAOX may be due to the extended treatment and/or a relatively moderate-risk-of-death CLP cohort. Long-term effect of mtAOX in abdominal sepsis appears different to sepsis/inflammation models arising from other body compartments.

## 1. Introduction

Sepsis is a deleterious clinical condition caused by a deregulated host response to infection associated with organ damage [1]. In immunocompetent individuals, sepsis provokes a robust systemic inflammatory response (which can coexist with concurrently developing immunosuppression). Various microbial, fungal, or viral components in the invaded host lead to a rapid, simultaneous release of pro- and anti-inflammatory mediators [2] and general activation of the innate/adaptive immunity. The acute phase of humoral and cellular response is accompanied by a rapid production of reactive oxygen species (ROS) [3]. Under physiological conditions, ROS are produced by different intra-/extracellular sources [4] with mitochondria as one of the main sites [5, 6]. Tightly regulated by cellular antioxidant mechanisms, at low-to-moderate concentrations, ROS have potent beneficial effects as an intrinsic part of various intracellular signaling pathways [5, 7, 8]. In contrast, increased ROS concentration (e.g., caused by depletion of regulatory antioxidants, inadequate antioxidative response [9], and/or altered ROS release) can elicit a chain reaction cascade that can damage the cells and tissues of the host [4]. This oxidative stress has been considered as one of the key contributors to the development of organ failure in sepsis and linked to a deregulation of mitochondrial function [10–12].

Numerous preclinical studies demonstrated clear benefits of an antioxidant-dependent inhibition of ROS production/release in different pathophysiological conditions [13, 14] including sepsis [15–19]. In contrast, clinical trials either failed to show any survival benefit in septic patients or were inconclusive [20–22]. In general, the incompatibility between preclinical and clinical studies (and among the clinical studies themselves) is due to numerous reasons including nonmatching models, erroneous study design, and/or nonspecific therapeutics. First, endotoxemia/LPS models are now considered inappropriate in recapitulating human sepsis syndromes [23]. Furthermore, due to a lack of clear dose-limiting toxicities, the dosages used in clinical trials are typically selected based on an “appeared feasible” subjective approach rather than preverified dose response studies [22]. For example, failure of a nonselective nitric oxide synthase inhibitor (46C88; L-NMMA acronym) phase 3 trial (versus phase 2) was partly attributed to the excessive L-NMMA dose and suboptimal selection of septic patients [24, 25]. Equally important is the element of therapeutic specificity: majority of studies applied nonselective antioxidants such as selenium, melatonin, ascorbic acid, n-acetylcystein, and *α*-tocopherol whose actions may be simultaneously too wide ranging and/or mistargeted.

Regarding the latter shortcoming, various conjugation protocols were developed (e.g., with lipophilic cations) to target the exogenous antioxidants directly to mitochondria. The best characterized to date is triphenylphosphonium (TPP) formulation [26]: it enables precise and uncorrupted cross-membrane delivery of antioxidants [27, 28] and has led to development of two very promising substances. SkQ1 is a novel, rechargeable plastoquinone derivative, which accumulates in the inner mitochondrial membrane due to its molecular charge and lipophilic properties [29, 30]. It shows antioxidant effects at much lower concentrations than other TPP conjugates, thus the “window” between anti- and prooxidant effects is larger [30]. MitoTEMPO is a piperidine-based nitroxide that works as a hydrophilic superoxide dismutase mimetic in the mitochondrial matrix. Positive preclinical results of SkQ1 and MitoTEMPO in various diseases including CLP [13, 31–35] and their strong immunomodulatory properties [36, 37] have been largely attributed to their high-target specificity.

Until now, MitoTEMPO (as mitochondria-specific ROS scavenger) was studied in the mouse CLP model for only a short term [35]. However, sepsis causes severe protracted sequelae and neither of those compounds has ever been tested in a clinically relevant sepsis model for their long-term effects. Hence, in this study, we investigated the influence of these two specific TPP-conjugated ROS scavengers, SkQ1 and MitoTEMPO, upon inflammatory response, organ function, and long-term outcome in a mouse model of polymicrobial acute sepsis originating from the abdominal compartment.

## 2. Materials and Methods

### 2.1. Animals

Female, 3-month-old, outbred CD-1 mice (Charles River Laboratories; *n* = 142) were used for all experiments (the exact *n*/group is specified in the legend to each figure). The genetic diversity of the CD-1 outbred strain (e.g., compared to BALB/c) is closer to patient diversity and therefore clinically more relevant. We chose sexually mature, healthy females to exclude confounding tangibles such as comorbidities and young/old age and to follow on the NIH recommendation to study females given their underrepresentation in preclinical intensive care research. Experiments 1 and 2 were performed in Ludwig Boltzmann Institute for Experimental and Clinical Traumatology (LBI), Vienna, Austria, and experiment 3 was performed in Institute of Surgical Research (ISR), University of Szeged, Faculty of Medicine, Szeged, Hungary. All mice were allowed to acclimatize to their new environment for at least one week after arrival. Mice were housed on a 12 h light-dark cycle with controlled temperature (21–23°C) and provided with standard rodent diet and water *ad libitum.*

### 2.2. Ethics Statement

All animal procedures were approved by the Viennese (Austria) Legislative Committee (Animal Use Proposal Permission number: 006596/2011/11) and the Hungarian Scientific Ethical Committee on Animal Experimentation (Animal Use Proposal Permission number: V./148/2013) All experiments were conducted according to the National Institutes of Health guidelines.

All mice were monitored by trained professionals at least three times per day and more whenever an animal's condition deteriorated [38, 39]. In order to avoid unnecessary suffering of CLP mice facing imminent death, we use in our laboratory a custom-developed scoring approach that combines the mouse clinical assessment scoring system (M-CASS) and sequential body temperature (BT) measurements. In brief, well-being and general condition of mice (i.e., fur, posture, mobility, alertness, weight, and startle reflex) were assessed every 12 hours (starting 12 h post-CLP), by assigning mice to one out of maximum three severity grades (i.e., 0, 1, or 2 points). Additionally, we measured inner BT. Mice were euthanized once the score indicated imminent death, that is, a score ≥ 8 and/or inability to trigger the startle reflex and/or BT < 28°C (recorded in at least two sequential measurements; the score sheet in Supplementary Figure 1 available online at https://doi.org/10.1155/2017/6412682). This approach is generally in line with the recent mouse M-CASS philosophy proposed by Lilley et al. [40]. The BT-based prediction of outcome we have developed in our laboratory is very precise (AUC = 0.94; [41]). Combination of the BT-based prediction with our custom-developed (M-CASS-like) scoring system prevents overliberal euthanasia decision-making, that is, allocation of potential survivors to the P-DIE group in survival studies.

### 2.3. Sepsis Model

Mice were subjected to cecal ligation and puncture (CLP) surgery according to the protocol by Wichterman et al. [42] with modifications described elsewhere [43]. We performed a medium severe CLP (18G needle) to reach an approximately 40% mortality (i.e., by day 28 post-CLP) that corresponds to mortality of human patients suffering from abdominal sepsis [44, 45]. Briefly, after opening the abdominal cavity via midline laparotomy, the cecum was exposed, ligated, and punctured twice (small amount of feces was extruded to ensure patency of the punctures). After repositioning of the cecum, the abdomen was closed with two single button sutures and Histoacryl® skin adhesive. Starting 2 hours post-CLP, all mice received subcutaneous broad-spectrum antibiotic therapy (25 mg/kg imipenem/cilastatin, Zienam®) and fluid resuscitation (1 ml Ringer's solution) with analgesia (0.05 mg/kg buprenorphine, Bupaq®) twice daily (in approx. 12 h intervals) for five consecutive days (experiment 1) or until the time point of euthanasia (in experiments 2 and 3). Five-day course of antibiotic coverage is routinely employed in CLP studies [46].

### 2.4. Study Design

#### 2.4.1. CLP Experiments

**Figure 1:**
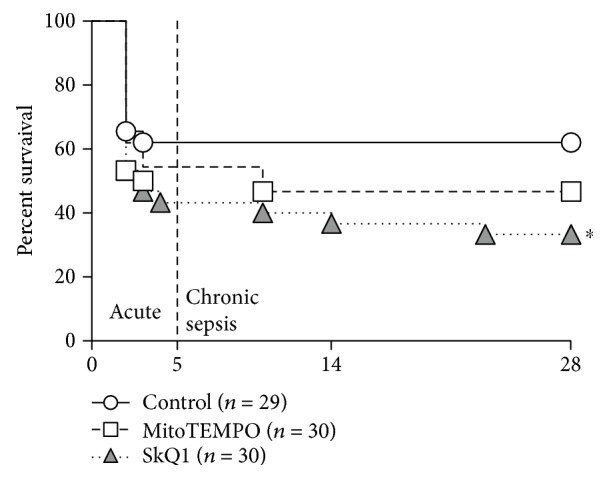
28-day survival of SkQ1-, MitoTEMPO-, and placebo-treated control mice. Mortality of treated and placebo mice subjected to CLP sepsis. Number of mice in each group listed in the legend (in brackets). SkQ1 versus control: ^∗^*p* = 0.03; MitoTEMPO versus control: *p* = 0.24.

The entire study was performed in three separate experimental blocks: experiment 1 as the main survival part (SkQ1 and MitoTEMPO; Figures 1(), 2(), 3(), 4(), and 5()) and follow-up experiment 2 (SkQ1 only; Figures 6() and 7(); both in LBI) and experiment 3 (SkQ1 only; Figure 8(); in ISR). To maximize reliability and minimize random effects in experiment 1, we conducted CLP in small groups of 15 mice per repetition (6 repetitions in total). Moreover, irrespective of location, all CLP surgeries were performed by the same operator (P.R.).

The follow-up experiments 2 and 3 had different sacrifice time points: at 48 h in experiment 2 and at 24 h post-CLP in experiment 3 (Figure 9() scheme). The time points were chosen based on the investigated endpoints: 48 h time point for bacterial load at the time of the maximal post-CLP mortality (experiment 2) and 24 h time point for potential mitophagy; the period closely preceding the most robust post-CLP deaths (experiment 3).

To ensure similar CLP severity between LBI (experiments 1 and 2) and ISR (experiment 3), we used BT profiling, that is, three (separate) pilot CLP runs were performed in ISR and the BT profiles of CLP mice (Supplementary Figure 1) were compared to the main CLP survival study (experiment 1). The third CLP run (i.e., experiment 3) showed nearly an identical BT profile to experiment 1 and was extended to reach the total *n* = 28.

#### 2.4.2. Treatment

All animals (total of *n* = 90 in experiment 1; *n* = 24 in experiment 2; and *n* = 28 in experiment 3; detailed *n*/group distribution in legends to figures) were randomly assigned to receive (in a blinded manner) either SkQ1 (5 nmol/kg), MitoTEMPO (50 nmol/kg), or placebo treatment (saline) via intraperitoneal injection at maximally five subsequent time points (i.e., 1, 12, 24, 36, and 48 h) post-CLP (see Figure 9 scheme for detailed description of experimental groups). For SkQ1, this amounts to 0.0062 mg/kg/d for the first two days and 0.0031 mg/kg/d for the third day. For MitoTEMPO, this amounts to 0.051 mg/kg/d for the first two days and 0.0255 mg/kg/d for the third day.

For all experiments, SkQ1/MitoTEMPO was dissolved in ethanol (96%) and diluted in saline in further steps. Freshly prepared dilutions were divided into aliquots (one for each treatment time point separately) and stored frozen at −80°C protected from light until use. The presence of SkQ1/MitoTEMPO molecules was verified on the first and the last days of experiments. The concentration of SkQ1 was tested spectrophotometrically as described in Antonenko et al. 2008 [30]. MitoTEMPO was measured with electron spin resonance spectroscopy as previously described [47].

We elected to administer the first antioxidant dose 1 h post-CLP to approach clinical relevance. We treated mice over the period of 48 h post-CLP given that it directly precedes/overlaps with the period of the most robust mortality in the acute CLP phase [48, 49]. Our treatment scheme is consistent with the one employed by Plotnikov et al. [13]. The selected antioxidant concentrations were adopted from Weidinger et al. [50]. Given that the effects of SkQ1 were predominantly examined in rats, we have compared the effects of increasing SkQ1 concentrations on the respiratory activity of mitochondria in liver homogenates from rats and mice. This was done to ensure that we remain within the nontoxic range of SkQ1 concentrations (Supplementary Figure 2).

In the follow-up experiments 2 and 3, SkQ1 (or placebo) was tested given that only SkQ1 treatment significantly exacerbated mortality in experiment 1. In experiment 2, mice received four injections (the last at 36 h post-CLP) and were sacrificed for peritoneal lavage and spleen collection. In experiment 3, mice received three injections (the last at 24 h post-CLP) and were sacrificed to collect the liver for mitophagy assessment (Figure 9 scheme). Irrespective of sacrifice, the identical protocol for treatment and sampling procedures was used in experiment 1 and both follow-up experiments 2 and 3.

Of note, in experiment 2, to generate Figure 6(a), septic mice were compared using the above-described M-CASS-like criteria, that is, using our custom-developed scoring system and BT monitoring; we retrospectively assigned mice into either predicted-to-die (P-DIE; inner BT ≤ 28°C) or predicted-to-survive (P-SUR; inner BT ≥ 35°C) group. For maximally conservative approach, all mice featured in Figure 6(a) met the objective [41] BT cut-off criteria (i.e., mice meeting the euthanasia threshold based solely on the clinical assessment ≥ 8 points were not included).

### 2.5. Blood Sampling

Repeated low-volume blood sampling was used in all groups reducing the total number of mice needed for the study [51]. Blood was collected immediately before CLP (baseline) and at 6, 24, 48, and 72 h thereafter in experiment 1 and for shorter periods (depending on the sacrifice time point) in experiments 2 and 3 (Figure 9 scheme).

Specifically, 30 *μ*l of blood was drawn by puncturing the facial vein (*vena submandibularis*) with a 23 gauge needle. Samples were then collected with a pipette rinsed with ethylenediaminetetraacetic acid (K_3_-EDTA) (diluted 1 : 50) and immediately diluted 1 : 10 in PBS. After centrifugation (1000 ×g, 5 min, 22°C), 270 *μ*l of plasma was removed and stored at −80°C until further analysis.

### 2.6. Abdominal Lavage

In experiment 2, after sacrifice (isoflurane anesthesia followed by cervical dislocation), we performed abdominal lavages with 5 ml cold PBS/3% fetal calf serum, using an adapted protocol from Ray and Dittel [52]. The collected cell suspension was deposited in tubes and kept on ice until further FACS analysis.

### 2.7. Complete Blood Count

After plasma was removed, we resuspended the remaining blood pellet with 180 *μ*l Cell-Dyn buffer with EDTA and a complete blood count with differential (erythrocytes, hemoglobin, platelets, white blood cells, neutrophil granulocytes, and lymphocytes) was performed with a Cell-Dyn 3700 counter (Abbott Laboratories, Illinois, USA) as previously described [51].

### 2.8. Cytokine and Chemokine Measurement

Interleukin (IL)-1*β*, IL-5, IL-6, IL-10, and IL-12p70, interferon (IFN)-*γ*, tumor necrosis factor (TNF)-*α*, macrophage inflammatory protein (MIP)-1*α*, chemokine ligand (KC; CXCL-1), and monocyte chemoattractant protein-1 (MCP-1) were analyzed from plasma samples according to the manufacturer's protocol using FlowCytomix™ Multiplex Kits (eBioscience, USA).

### 2.9. Metabolic and Organ Function/Cell Injury

Plasma levels of urea, glucose, lactate dehydrogenase (LDH), and alanine transaminase (ALT) were analyzed with Cobas c111 analyzer (Roche, Switzerland). The lower detection limit for urea was 3 mg/dL, for glucose 1.98 mg/dL, for LDH 10 U/L, and for ALT 2 U/L. The inner body temperature was measured (at least twice per 24 h) using a Fluke 52 Series II thermometer (Fluke, USA) with a rectal probe.

### 2.10. Nitrate/Nitrite (NOx) Measurement

The Nitric Oxide Analyzer (NOA) (Nitric Oxide Analyzer Sievers 280i, GE Analytical Instruments, USA) consisted of a glassware system and an electronic detection unit. Plasma samples were injected through a septum into the glass vessel, where NO species were converted selectively to NO_(g)_ by a redox active reagent (VCl_3_ for total NO). Following the reduction step, NO molecules are carried to the reaction chamber of the detection unit by N_2_, an inert gas which was constantly purged through the mixture during measurements. Inside the reaction chamber, NO from the gas phase reacted rapidly with O_3_ to form NO_2_^∗^ in an excited state (NO + O_3_ → NO_2_^∗^ + O_2_). As the excited e^−^ returned to its ground state, a photon was emitted and detected as chemiluminescence (*hv*) (NO_2_^∗^ → NO_2_ + *hv*). Emitted light was detected and amplified by a photo multiplier tube (PMT) to generate an electric signal. The detection limit for NO and related species in liquid samples was ~1 pmol.

### 2.11. Flow Cytometry

Peritoneal lavage fluid samples were kept in 50 ml Falcon tubes on ice and were processed within 1 hour of collection. After red blood cell lysis, the cell suspension was filtered through a 70 *μ*M cell strainer (BD, Bedford, MA, USA) to singularize the cells, washed with PBS, and adjusted to the recommended cell count for staining. A total of 100 *μ*L of the suspension was then transferred to FACS tubes (Beckman Coulter, Brea, CA, USA) and incubated with fluorophore-conjugated antibodies on ice for 30 min. If not indicated otherwise, all antibodies were purchased from eBioscience (Vienna, Austria). Leukocyte subsets in the lavage fluid were identified by morphology and the positivity/negativity for the following antigens: presence of the integrin subunit CD11b (-FITC conjugate) and the granulocyte cell surface determinant Ly6-G (Gr-1, APC-conjugate) as well as the absence of the macrophage adhesion receptor F4-80 (PE-conjugate) was used to determine neutrophil populations. CD11b/F4-80-positive and Ly6-G-negative subsets were identified as macrophages. Cell suspensions were washed with PBS and were measured on a FC-500 flow cytometer (Beckman Coulter, Brea, CA, USA). A minimum of 1 × 10E4 intact cells were recorded and analyzed in FlowJo software (FlowJo LLC., Ashland, Oregon); Leukocyte subsets were displayed as % of intact cells.

### 2.12. Bacterial Growth Assessment

The spleens were obtained immediately after sacrifice under sterile conditions following the protocol of Barquero-Calvo et al. [53] allowing small modifications. We used nine parts of PBS containing 0.1% Tween 20 per g of spleen (dilution 1 : 10), assuming that the volume of 1 g of spleen corresponds to 1 ml of PBS (e.g., 0.5 g of spleen and 4.5 ml of PBS 0.1% Tween 20). Isolated spleens were homogenized using pellet pestles (Dstroy-S-15, Biozym, Oldendorf). After centrifugation at 13000 rpm for 10 minutes, supernatant was removed and serially diluted with PBS for subsequent plating on lysogeny broth (LB) agar plates (Miller, USA) and incubation overnight at 37°C. Colony-forming units were counted after 24 h.

### 2.13. Western Blotting

Mice were euthanized at 24 h post-CLP, and liver samples were immediately snap-frozen in liquid nitrogen and stored at −80°C for further analysis. All analyses were performed in Jena University Hospital (Germany). Total protein content was isolated from the frozen tissue samples by a protein isolation buffer pH 7.5 (10 mM Tris, 250 mM saccharose, 1 mM EDTA, 1% phosphatase-inhibitor cocktail, and 1% protease inhibitor 2) using a dounce homogenizer (Kimble Chase, Mexico). Homogenates were centrifuged at 900*g* for 10 min at 4°C to obtain the post nucleus fraction, followed by 100,000*g* for 1 h at 4°C to separate the cytosolic and microsomal fractions. Protein content in the post nucleus supernatant was determined using the bicinchoninic acid (BCA) assay (Sigma Aldrich, Hamburg, Germany). Fractions were used as indicated. 20 µg protein was loaded on a sodium dodecyl sulfate (SDS) gel and transferred to a polyvinylidenfluorid (PVDF)-membrane (Roth, Karlsruhe, Germany). Membranes were incubated with 5% N,O-bis(trimethylsilyl)acetamid (BSA) or skim milk (Sigma-Aldrich, Hamburg, Germany) in tris-buffered saline with Tween20 (TBST) for 1 h at room temperature. Primary antibodies in 5% BSA or skim milk were incubated on a shaker overnight at 4°C. Horseradish peroxidase (HRP)-conjugated secondary antibodies (1 : 5000) were incubated for 1 hour at room temperature. The following primary antibodies were used: anti-LC3B, TOM20, cytochrome C (Cell Signaling Technology®, Danvers, USA), and p62 or anti-*β*-actin (Abcam®, Cambridge, UK). Detection of the secondary antibody by chemiluminescence was performed using HRP substrate (Immobilon Western HRP Substrat, Merck Millipore, Darmstadt, Germany) by the LAS3000 (GE Healthcare, Frankfurt, Germany). Protein content was quantified using software *ImageJ* (National Institutes of Health, Bethesda, MD, USA). *β*-Actin or Coomassie-stained gel served as loading control to calculate the differences in the protein expression between the groups. To quantify the level of autophagy, the LC3B-II : LC3B-I ratio was calculated based on a densitometric analysis and normalized to *β*-actin.

### 2.14. Statistical Analysis

The Kaplan-Meier method was used to plot and compare the 28-day survival in experiment 1. All other data sets were tested for normality before further analysis. Abnormally distributed data sets were log-transformed to achieve normal distribution. Comparisons among the three groups were made using one-way ANOVA and Bonferroni's Multiple Comparison Test for the parametric data and Kruskal-Wallis Test and Dunn's Multiple Comparison Test for the nonparametric data. To compare the SkQ1 with the placebo group, we used the unpaired Student *t*-test for parameters with parametric distribution or the Mann–Whitney Test for parameters with nonparametric distribution. The level of significance was set at *p* < 0.05. All statistical analyses and graphs were made with GraphPad (San Diego, USA).

## 3. Results

### 3.1. Application of Mitochondrial ROS Scavengers Exacerbated Mortality

Survival was monitored for 28 days post-CLP. To be consistent with clinical severity of abdominal sepsis, mice were subjected to a medium-severe CLP with 38% mortality (average of six CLP repetitions). Neither MitoTEMPO nor SkQ1 exerted a protracted survival benefit. Conversely, SkQ1 treatment exacerbated mortality to 67% (*p* = 0.03) by day 28 (Figure 1). This negative effect was consistent in all six CLP repetitions (Supplementary Figure 3); it was apparent immediately in the acute phase (i.e., within 1–5 days post-CLP; in four CLP repetitions) and continued into the chronic phase until the end of the observation period. MitoTEMPO effect was inconsistent among individual repetitions (not shown) and did not reach statistical significance (*p* = 0.24; Figure 1).

### 3.2. Application of Mitochondrial ROS Scavengers Did Not Modulate the Inflammatory Response to Polymicrobial Sepsis

**Figure 2:**
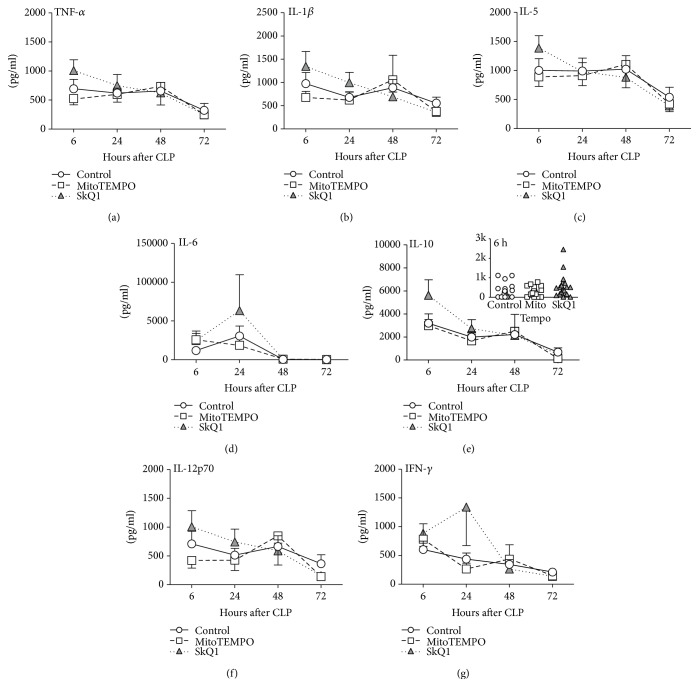
Comparison of circulating cytokines between SkQ1-, MitoTEMPO-, and placebo-treated control mice. (a–g) Plasma levels of TNF-*α*, IL-1*β*, IL-5, IL-6, IL-10, IL-12p70, and IFN-*γ* in treated mice (SkQ1 or MitoTEMPO) at 6, 24, 48, and 72 h post-CLP were compared to the placebo group (CLP + NaCl). For (a–g): at 6 h: control *n* ≥ 19, SkQ1 *n* = 19, MitoTEMPO *n* = 19; at 24 h: control *n* = 19, SkQ1 *n* = 17, MitoTEMPO *n* = 15; at 48 h: control *n* = 13, SkQ1 *n* = 12, MitoTEMPO *n* = 10; at 72 h: control *n* = 13, SkQ1 *n* = 11, MitoTEMPO *n* = 9. Data points shown as mean ± SEM. The exemplary IL-10 inset depicts scatter plot of all groups at 6 h post-CLP time point. 2 k (for brevity) corresponds to 2000 pg/ml.

We examined a comprehensive set of circulating cytokines that are typically upregulated in both human and mouse sepsis [54, 55]. In general, CLP induced an increase of all cytokines measured; the strongest concentration was recorded for IL-6, MCP-1, CXCL-1, and MIP-1*α* at 24 h (Figures 2() and 3()). None of those post-CLP increases was further significantly modified by either SkQ1 or MitoTEMPO.

Following acute sepsis, a strong rearrangement of cell populations occurs. We found out that neither SkQ1 nor MitoTEMPO affected the CLP-induced lymphopenia (at 24 h: placebo 1.2 pg/ml versus SkQ1 1.1 pg/ml versus MitoTEMPO 0.9 pg/ml) and neutrophilia (at 24 h: placebo 0.4 pg/ml versus SkQ1 0.4 pg/ml versus MitoTEMPO 0.3 pg/ml) when compared to the placebo group (Supplementary Figure 5).

### 3.3. Application of Mitochondrial ROS Scavengers Did Not Change Organ Function/Injury and Metabolic Parameters

**Figure 4:**
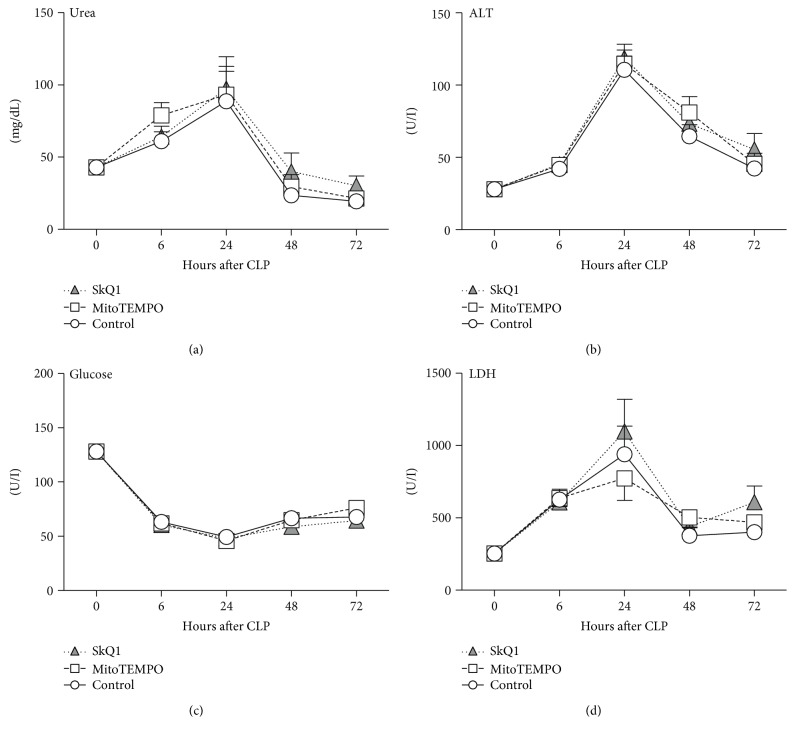
Comparison of organ function parameters between SkQ1-, MitoTEMPO-, and placebo-treated control mice. Plasma levels of treated mice (SkQ1 or MitoTEMPO) of (a) urea, (b) ALT, (c) glucose, and (d) LDH in mice at baseline and post-CLP were compared to the control group (CLP + placebo). For (a–d): at BL *n* = 20; at 6 h control *n* = 20, SkQ1 *n* = 19, MitoTEMPO *n* = 19; at 24 h control *n* = 19, SkQ1 *n* = 17, MitoTEMPO *n* = 17; at 48 h control *n* = 13, SkQ1 *n* = 12, MitoTEMPO *n* = 10; at 72 h control *n* = 13, SkQ1 *n* = 11, MitoTEMPO *n* = 9. Data points shown as mean ± SEM.

Functional tissue damages are hallmarks of sepsis pathogenesis [9]. We followed temporal fluctuations of glucose (GLU), alanine aminotransferase (ALT), lactate dehydrogenase (LDH), and urea in septic mice after CLP. In general, all groups displayed similar trajectories at all time points (Figure 4). Specifically, ALT, LDH, and urea peaked in all treatment groups at 24 h (ALT: placebo 111 U/l versus SkQ1 119 U/l versus MitoTEMPO 115 U/l; LDH: placebo 940 U/l versus SkQ1 1097 U/l versus MitoTEMPO 773 U/l; urea: placebo 89 mg/dl versus SkQ1 97 mg/dl versus MitoTEMPO 93 mg/dl), while blood glucose decreased by approx. 63% (BL: 128 mg/dl versus 24 h: 49 mg/dl) in the placebo group. Hypoglycemia persisted until 72 h post-CLP (placebo 68 mg/dl versus SkQ1 64 mg/dl versus MitoTEMPO 76 mg/dl), while the other parameters recovered.

### 3.4. Application of Mitochondrial ROS Scavengers Did Not Affect NOx Concentration in Plasma

**Figure 5:**
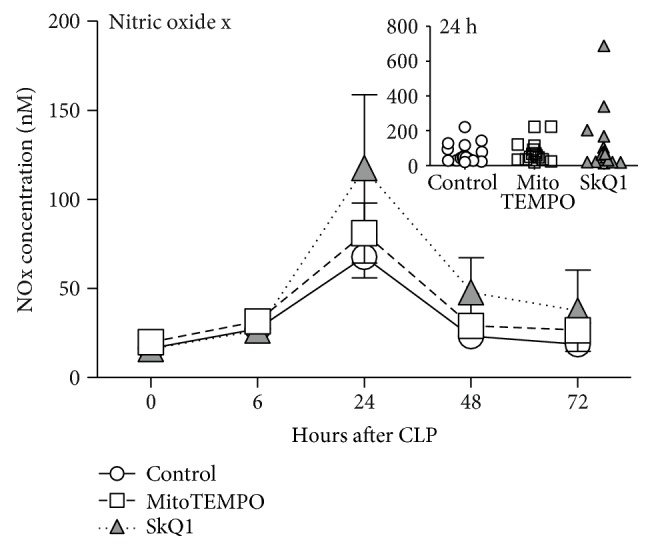
NOx concentration in the blood plasma. All forms of NO (NOx) in plasma were measured at BL (0 h), 6, 24, 48, and 72 h post-CLP. At BL: control *n* = 20, SkQ1 *n* = 20, MitoTEMPO *n* = 20; at 6 h: control *n* = 20, SkQ1 *n* = 19, MitoTEMPO *n* = 19; at 24 h: control *n* = 19, SkQ1 *n* = 17, MitoTEMPO *n* = 15; at 48 h: control *n* = 13, SkQ1 *n* = 12, MitoTEMPO *n* = 10; at 72 h: control *n* = 13, SkQ1 *n* = 11, MitoTEMPO *n* = 9. Data points shown as mean ± SEM. The inset depicts scatter plot of all groups at 24 h post-CLP time point.

Nitric oxide (NO) and its metabolized forms nitrite (NO_2_^−^) and nitrate (NO_3_^−^; both referred to as NOx) play a central role in inflammation and correlate with severity of sepsis. We measured NOx concentration in plasma at BL, 6, 24, 48, and 72 h post-CLP (Figure 5); there were no significant intergroup NOx changes. Similar to the organ function parameters, NOx peaked at 24 h post-CLP (approx. 4-fold higher than baseline).

### 3.5. SkQ1 Treatment Did Not Impair the Bacterial Clearance in the Spleen

**Figure 6:**
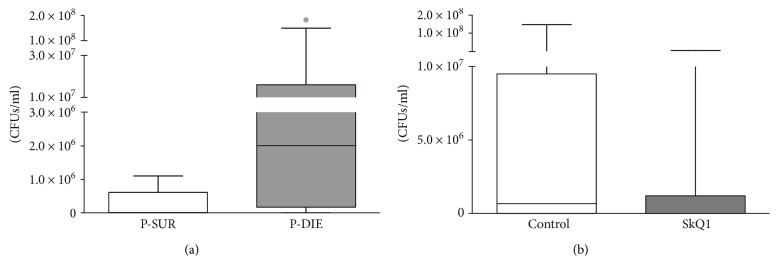
Bacterial load in the spleen. Number of colony-forming units (CFUs) per ml spleen homogenate. (a) Mice that were predicted-to-die (P-DIE; *n* = 7) versus predicted-to-live (P-SUR; *n* = 11). Prediction of outcome was performed retrospectively based on the body temperature recordings taken before sacrifice time point at 48 h post-CLP. (b) Placebo-treated (*n* = 7) versus SkQ1-groups (*n* = 10). Data as (min–max) box-and-whiskers plots ^∗^*p* < 0.05.

Because of the consistent survival deterioration after SkQ1 (but not MitoTEMPO), we only compared the SkQ1 and placebo groups in experiment 2. First, to verify the bacterial clearance, we determined colony-forming units (CFUs) in the spleen homogenates. Mice that were predicted to die post-CLP had approximately 100-fold more CFUs in the spleen than mice that were predicted to survive CLP (Figure 6(a); *p* = 0.03). However, this effect was not related to antioxidant treatment (Figure 6(b)).

### 3.6. SkQ1 Treatment Did Not Change Macrophage or Granulocyte Counts from Peritoneal Lavages

**Figure 7:**
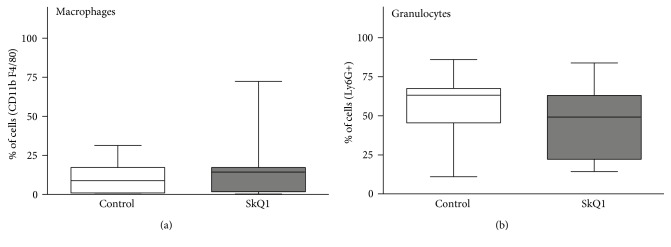
Distribution of macrophages and neutrophils in peritoneal lavages of the placebo-treated control group versus SkQ1-treated group. Flow cytometry determination of macrophages and neutrophils, displayed as percent of intact measured cells. Placebo *n* = 7, SkQ1 *n* = 9. Data as (min–max) box-and-whiskers plots.

In experiment 2, to identify potential treatment-related differences in the abdominal leukocyte populations, we performed peritoneal lavages at 48 h post-CLP and analyzed cells by FACS. In general, the cell counts were similar between both groups (Figures 7(a) and 7(b)): macrophages (CD11b+/F4/80+) reached 11% in placebo and 16% in SkQ1, while granulocytes (Ly6G+) reached 56% in placebo and 45% in SkQ1.

### 3.7. SkQ1 Treatment Did Not Modify the Mitochondrial Autophagy

**Figure 8:**
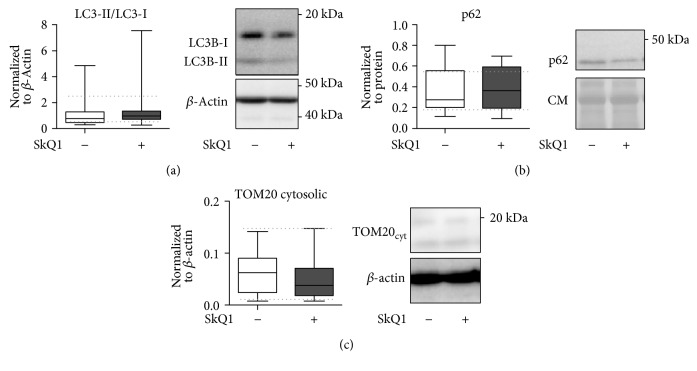
Comparison of mitophagy and mitochondrial integrity in the liver of placebo-treated control versus SkQ1-treated groups at 24 h post-CLP. CLP mice received a total of three SkQ1/placebo injections before sacrifice at 24 h. All panels display densitometric analysis of the Western blot assay as well as representative Western blots. (a) LC3-II-to-LC3-I ratio (normalized to actin), (b) p62 (normalized to total protein), and (c) cytosolic TOM-20 release (normalized to actin). Total number of CLP mice loaded on three different gels: SkQ1 *n* = 16; control (placebo) *n* = 12. Data as (min–max) box-and-whiskers plots. Dotted lines indicate upper/lower standard deviation calculated based on eight healthy control mice (no CLP, no treatment) that were analyzed together with the CLP mice. CM: Coumassie stained gel.

**Figure 9:**
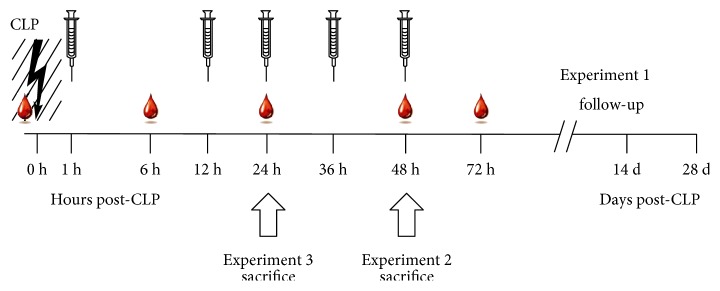
Schematic illustration of treatment, blood/tissue sampling, and monitoring. The entire study was divided into three experimental parts that followed the same treatment protocol with specific modifications described below and depicted graphically in the figure. 3-month-old female mice were subjected to polymicrobial CLP sepsis. Mice were subjected to low-volume repetitive blood sampling (30 *μ*l blood volume/sampling; time points indicated by a single blood drop) maximally five times: At baseline (BL, immediately before CLP) 6 h, 24 h, 48 h, and 72 h post-CLP. Maximally, five intraperitoneal injections with either SkQ1, MitoTEMPO, or placebo treatment were conducted at 1 h, 12 h, 24 h, 36 h, and 48 h post-CLP (indicated by syringes). In experiment 1: mice (*n* = 90) were monitored for 28 days (all five blood collections and treatment injections performed). In experiment 2: mice (*n* = 24) were sacrificed at 48 h post-CLP (four blood collections and treatment injections performed). In experiment 3: mice (*n* = 28) were sacrificed at 24 h post-CLP (three blood collections and treatment injections performed). White block arrows indicate sacrifice time point for experiments 2 and 3.

Finally, we investigated mitophagy and integrity of mitochondria in the liver (as a relatively sensitive organ) to establish whether SkQ1 exerted a direct detrimental effect upon this organelle. Western blot analysis of key proteins involved in autophagy (i.e., LC3B, p62) [56], mitochondrial membrane integrity (i.e., TOM20; Figures 8(a), 8(b), and 8(c)), and apoptosis (i.e., cytochrome C; Supplementary Figure 5) was performed. Compared to CLP mice treated with placebo, SkQ1 failed to reveal any modulation in the expression of those markers in the liver at 24 h post-CLP. This implies that the SkQ1-induced exacerbation of CLP mortality was not caused by direct mitochondrial damage.

## 4. Discussion

Recently, Sepsis-3 guidelines redefined sepsis as a “life-threatening organ dysfunction caused by a dysregulated host response to infection” [1]. ROS production/release is thought to play a major part in the progression of organ failure and mitochondrial dysfunction [12, 57]. Therefore, we aimed to improve the outcome of mice with polymicrobial sepsis by selectively inhibiting ROS at one of its key formation sites, the mitochondrion. Surprisingly, none of the tested antioxidants demonstrated any benefits, while SkQ1 markedly increased CLP mortality.

Best to our knowledge, this is the only study demonstrating a negative long-term impact of targeted mitochondrial antioxidant scavenging (i.e., by SkQ1) upon abdominal sepsis survival. The majority of rodent studies showed benefits of SkQ1 and/or MitoTEMPO in acute inflammatory conditions including pyelonephritis [13], gastritis [58], cardiomyopathy [31], SIRS [59], and sepsis [35]. Similar was true in a rat model of pneumosepsis [60] and endotoxemia [14]. Antioxidant (not mitochondria targeted) treatment did not improve survival only in three mouse studies, acute pancreatitis [61], pancreatic cancer [62], and CLP [63] models. While surprising, our protracted study design (Supplementary Figure 3) allows postulating that the reduction of survival due to SkQ1 was not a coincidental observation.

First, we examined two main elements of the systemic CLP response, inflammation, and organ function, to discern the deleterious mechanism(s). We repeatedly measured over twenty relevant parameters in mice enrolled in the survival study. This ensured maximal synchrony between the end-outcome effect and detection of a potential mechanism-of-action. CLP-induced dysregulation of the inflammatory and organ function system was consistent with data reported by us [64, 65] and others [66]. Surprisingly, we failed to observe any additional consistent changes caused by the SkQ1 and/or MitoTEMPO. This is suggestive of two things. First, the increased mortality in the SkQ1 mice did not appear to be caused by an evidently exacerbated early systemic inflammatory response, especially given that the four-day cytokine trajectories preceded/overlaid with the period of the highest acute mortality (days 1–3 post-CLP). Second, hepatocyte damage and/or impaired kidney function as well as dysregulation of carbohydrate metabolism was not a culprit either. The latter, however, does not exclude potential late derangements occurring in those and other organs (e.g., heart, lungs, and intestines) and/or systems (e.g., microcirculation, coagulation) that we did not examine. The protracted readout of the CLP-induced NOx fluctuations in the blood was consistent with the above changes. Similar to other works [67], the post-CLP peak of circulating nitrate/nitrite occurred in our study at 24 h time point but it was not further modified by antioxidants.

Next, we tested whether SkQ1 modulated the abdominal cellular characteristics and/or impeded bacterial clearance. We performed two-tier retrospective comparison: (1) treatment versus control as well as (2) predicted-to-die versus predicted-to-live using the body temperature-based prediction of outcome [41]. Such an approach allows more precise verification of the potential treatment effect upon the lethal phenotype. Our data show that compared to (predicted-to-be) survivors, predicted-to-die mice had much more pronounced bacterial load in the spleen. Yet again, the placebo treatment comparison revealed that SkQ1 neither altered the abdominal recruitment of phagocytes nor interfered with the bacterial clearance.

Impaired mitochondrial function was not always detected in CLP models, particularly in rodents [68]. Yet, the absence of a visible mitochondrial dysfunction in such cases was explained by activation of the effective autophagy process and removal of nonfunctional mitochondria. Thus, the autophagy appeared to us as a very sensitive sensor of the potential impairment of mitochondrial function. The final experiment 3 showed, however, that SkQ1 treatment was not by itself harmful to mitochondria (in the liver) given that neither autophagy (i.e., mitophagy) nor mitochondrial membrane integrity was altered by SkQ1 at the administered dose. This is in line with the nontoxicity assessment of SkQ1 in liver homogenates of healthy mice and rats (Supplementary Figure 2).

This leaves us with a negative effect of SkQ1 upon sepsis survival and insufficient understanding what this effect can be attributed to. There are several ways to explain the absence of the expected antioxidant benefits as well as the uncertain mechanism-of-action behind the worsened mortality. First, a proper dose fine tuning of the antioxidants appears important. For example, at micromolar concentrations, cationic quinone derivatives have a strong pro- not antioxidant effect [29]. Thus, it is unlikely that a higher dose would have lessened (reversed) the observed adverse SkQ1 effect. We rather consider inapt treatment timing and/or relatively low-risk-of-death sepsis CLP cohort as culprits. Specifically, we used a repeated treatment to cover the first two post-CLP days, the phase with the most frequent acute sepsis mortality. It seems now that such a blanket coverage might have been too aggressive and/or wide ranging, especially in a nonoverly lethal sepsis environment of our study. A minimal threshold of mitochondrial ROS (mtROS) is key for maintaining intracellular defense signaling pathways [69, 70] to enable, for example, a proper response to bacterial invasion [71]. Although we did not see a post-SkQ1 exacerbation of microbial load in the spleen, a complete blockage of mtROS (not detrimental to mitochondria themselves) could have impeded bacterial clearance at other sites. Likewise, blanket mtROS scavenging could have deregulated transcription of protective hypoxia inducible factors [72, 73] that we did not examine. Of note, there is an emerging role of cell-free hemoglobin in sepsis-induced organ failure [74]. For example, the recent clinical trial showed that blocking ferryl hemoglobin with acetaminophen improved sepsis outcome [75]. Thus, in addition to mtROS, rich amounts of ROS are generated by different intra/extracellular sources such as NADPH-oxidase, myeloperoxidase, and ferryl hemoglobin. Future studies need to explain precise site- and cell-specific roles of oxidative stress in sepsis. Of note, recent antioxidant treatment using N-acetylcystein and butylated hydroxyanisole (unspecific ROS scavengers) improved survival of mice with specific deletion of the iron sequestering protein ferritin heavy chain (FTH); restoration of hepatic glucose production was identified as the key mechanism of this benefit [76]. Given that our CLP mice did not enter a prolonged (potentially deadly) hypoglycemia, this beneficial effect would not have applied.

The two recent sepsis studies offer some relevant clues regarding the antioxidant treatment timing/dosage and model severity [13, 63]. At first, CLP mice were given a single-bolus coenzyme Q10 (strong mitochondria-targeted antioxidant) at 5 h and 20 h post-CLP. Only the latter attenuated organ injury and (insignificantly) improved survival [58]. The authors also noted that when sham (also control) mice were treated with coenzyme Q10, their injury score doubled (versus untreated sham/control) and a (statistically insignificant) decline in survival occurred in sham + coenzyme Q10 mice. This indicates a predilection for detrimental effects of coenzyme Q10 in a nonlethal milieu. The second study was performed in rats with acute pyelonephritis (APN) who benefited from SkQR1 (we reproduced their treatment protocol) [13]. There are four major differences between that study and ours: cumulative dosage (500 versus 25 nmol/kg), species (rat versus mouse), origin of sepsis (urosepsis versus abdominal), and the infection severity. We consider the latter two elements as essential. First, the rat APN model displayed approx. 70% while our model reached a total 40% mortality by day 28. We believe that treatment benefits are much more likely to occur in a lethal versus moderate severity. For example, inhibition of murine leukotriene B4 synthesis improved CLP survival in severe sepsis [77], while the same agent worsened survival by 50% in a sublethal CLP [78]. We recently showed that only CLP mice predicted-to-die benefited from dexamethasone [49], whereas PAI-1 inhibition exacerbated CLP mortality in mice that were predicted to survive [79]. A similar response scenario cannot be ruled out in our study given that on average surviving mice outweighed those with the lethal CLP phenotype as opposed to the (beneficial) coenzyme Q10 treatment (65% mortality) [63], MitoTEMPO mouse experiment (60% mortality), and SkQR1 APN rat study [13]. Likewise, in the recent SIRS study, 48 h pretreatment with SkQ1 improved mouse survival in a lethal but not in 50% mortality setup [59].

The negative effect of SkQ1 can be also attributed to the specificity of CLP; SkQ1 at the identical (pretreatment) dose was protective in the LPS rat model [50]. CLP only reproduces polymicrobial human sepsis of abdominal origin, and it cannot be reflexively extended to other sepsis types [2]. SkQ1 was never tested in CLP, and it is plausible that its negative effects may arise only in sepsis originating from this particular body compartment. Of note, our study monitored survival for 28 days. Although advised in preclinical sepsis [2], a protracted followup has never been tested before for targeted antioxidant treatment in sepsis. Had we used a 7-day observation, the SkQ1-induced effect would have been statistically insignificant.

There are several limitations in our study. First, only placebo versus verum CLP mice were compared and sham controls were not employed. Second, we did not assess the mitochondria-and/or cell-derived ROS. Third, mitophagy assessment was limited to a single (i.e., 24 h post-CLP) time point. Finally, CLP experiments were performed at two different locations (i.e., LBI and ISR) and the potential influence of macrobiome fluctuations was not verified.

It is also possible that the rodent CLP model is not an appropriate testing model for antioxidant therapies given the overall higher resistance of rodents to trauma/infection [2] and the qualitative difference in ROS release [80] under infectious and/or endotoxic stimuli (rodents versus humans). Thus, the sudden ROS release might not be as damaging in septic rodents as it is in patients. Long-term mouse studies using pneumosepsis, CASP, and candidiasis models as well as testing the mitochondria-specific antioxidants in various sepsis severity protocols will verify whether the negative effect observed here can be attributed to the CLP model itself, species used, the magnitude of the sepsis insult, or combination thereof. Finally, our study can be also considered as cautionary; it suggests that in certain severe inflammatory states, mtROS scavenging can be therapeutically inefficient and/or deleterious.

## Supplementary Material

Supplementary Fig. 1. Score sheet for a custom-developed mouse clinical assessment scoring system. The well-being of mice is assessed based on six endpoints every 12 hours (beginning at 12h post-CLP); tree severity grades (i.e. 0, 1 or 2 points) are assigned. Euthanasia is indicated when: score ≥8 and/or by inability to trigger the startle reflex and/or BT<28°C (recorded in at least two sequential measurements). Supplementary Fig. 2. Comparison of body temperature (BT) profiles in CLP experiments performed in two different laboratories. BT profile was used as a surrogate of outcome to optimally standardize the magnitude of CLP severity between the surgery performed in Ludwig Boltzmann Institute (LBI; Vienna, Austria) and Institute of Surgical Research (ISR), University of Szeged (Hungary). Mice underwent three CLP runs at ISR and their BT profiles (at 6h, 12h and 24h) were compared to the BT profile (at 6 and 24h post-CLP) of the CLP mice enrolled in the survival study performed in LBI (solid line/dot). ISR-CLP #3 was designated as the best match and expanded to n⁼28 for further mitophagy analysis. LBI-CLP n⁼90-78; ISR-CLP#1 n⁼28-16; ISR-CLP#2 n⁼25-13; ISR-CLP#3 n⁼27-26. Data points shown as mean±SEM. Supplementary Fig. 3. Effect of SkQ1 treatment on the state 3 mitochondrial respiration in the rat and mouse liver homogenates. State 3 respiration was measured in mouse and rat liver homogenates subjected to SkQ1 in the range of concentrations from 0 to 108.5nM. n⁼4/each species. Data points shown as mean±SD. Dotted lines indicate either the single (5nM) or cumulative (25nM) SkQ1 dose administered to CLP mice in the main survival study (Experiment 1). The rat data serve as species comparison. Supplementary Fig. 4. Visualization of outcome for each individual CLP run. CLP was performed in six independent reiterations with 14-15 mice at each repetition (typically 5 mice/each group; the precise n indicated on each panel). Statistical assessment of outcome was performed on the combined data set (Fig. 2). Supplementary Fig. 5. Trajectory of white blood cells (WBC; A), lymphocyte (LYM; B) and neutrophil (NEU; C) counts for SkQ1, MitoTEMPO and placebo mice (control). For A-C: at BL n⁼50; at 6h control n⁼20, SkQ1 n⁼25, MitoTEMPO n⁼23; at 24h control n⁼23, SkQ1 n⁼23, MitoTEMPO n⁼22; at 48h control n⁼18, SkQ1 n⁼15, MitoTEMPO n⁼15; at 72h control n⁼18, SkQ1 n⁼14, MitoTEMPO n⁼15. Data points shown as mean +/- SEM. Supplementary Fig. 6. Assessment of Cytochrome C release in the liver of placebo-treated control vs. SkQ1-treated group at 24h post-CLP. CLP mice received total of three SkQ1/placebo injections before sacrifice at 24h. Data is shown as densitometric analysis of the Western blot from cytosolic. Total number of CLP mice loaded on three different gels: SkQ1 n⁼16; control (placebo) n⁼12. Data as (min-to-max) box-and-whiskers plots. Dotted lines indicate upper/lower standard deviation calculated based on eight healthy control mice (no CLP, no treatment) that were analyzed together with the CLP mice.CM:.coumassie stained gel.

## Figures and Tables

**Figure 3:**
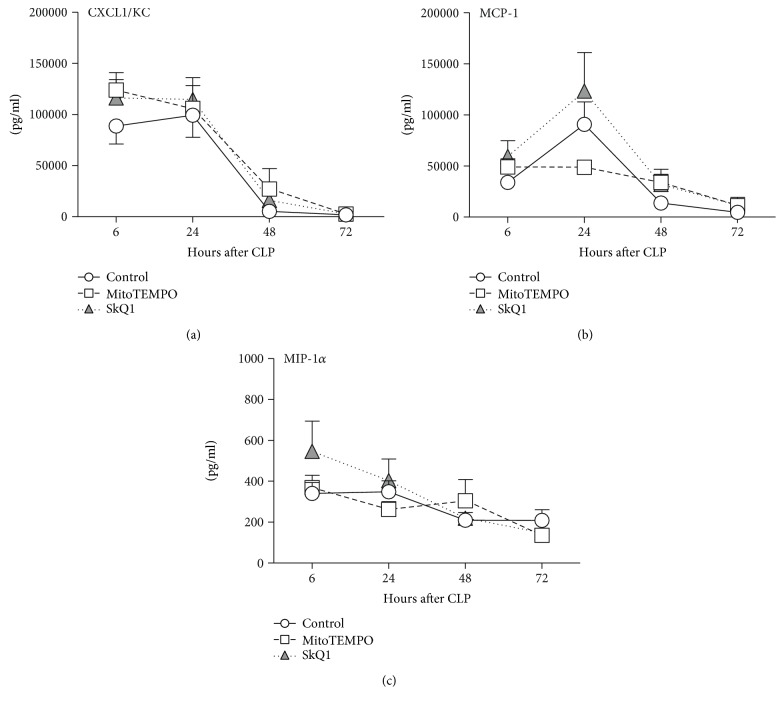
Comparison of circulating chemokines between SkQ1-, MitoTEMPO-, and placebo-treated control mice. (a–c) Plasma levels of CXCL1, MCP-1, and MIP-1*α* in treated mice (SkQ1 or MitoTEMPO) at 6, 24, 48, and 72 h post-CLP were compared to the placebo group (CLP + NaCl). For (a–c): at 6 h: control *n* = 19, SkQ1 *n* = 19, MitoTEMPO *n* = 19; at 24 h: control *n* = 19, SkQ1 *n* = 17, MitoTEMPO *n* = 15; at 48 h: control *n* = 13, SkQ1 *n* = 12, MitoTEMPO *n* = 10; at 72 h: control *n* = 13, SkQ1 *n* = 11, MitoTEMPO *n* = 9. Data points shown as mean ± SEM.
