# Supplementary material for: Mitochondria-Targeted Antioxidants SkQ1 and MitoTEMPO Failed to Exert a Long-Term Beneficial Effect in Murine Polymicrobial Sepsis
Source: Oxid Med Cell Longev. 2017 Sep 19;2017:6412682. doi: 10.1155/2017/6412682 (PMC5625755; doi:10.1155/2017/6412682)
Supplement: Supplementary file 1 — Supplementary Fig. 1. Score sheet for a custom-developed mouse clinical assessment scoring system. The well-being of mice is assessed based on six endpoints every 12 hours (beginning at 12h post-CLP); tree severity grades (i.e. 0, 1 or 2 points) are assigned. Euthanasia is indicated when: score ≥8 and/or by inability to trigger the startle reflex and/or BT<28°C (recorded in at least two sequential measurements). Supplementary Fig. 2. Comparison of body temperature (BT) profiles in CLP experiments performed in two different laboratories. BT profile was used as a surrogate of outcome to optimally standardize the magnitude of CLP severity between the surgery performed in Ludwig Boltzmann Institute (LBI; Vienna, Austria) and Institute of Surgical Research (ISR), University of Szeged (Hungary). Mice underwent three CLP runs at ISR and their BT profiles (at 6h, 12h and 24h) were compared to the BT profile (at 6 and 24h post-CLP) of the CLP mice enrolled in the survival study performed in LBI (solid line/dot). ISR-CLP #3 was designated as the best match and expanded to n⁼28 for further mitophagy analysis. LBI-CLP n⁼90-78; ISR-CLP#1 n⁼28-16; ISR-CLP#2 n⁼25-13; ISR-CLP#3 n⁼27-26. Data points shown as mean±SEM. Supplementary Fig. 3. Effect of SkQ1 treatment on the state 3 mitochondrial respiration in the rat and mouse liver homogenates. State 3 respiration was measured in mouse and rat liver homogenates subjected to SkQ1 in the range of concentrations from 0 to 108.5nM. n⁼4/each species. Data points shown as mean±SD. Dotted lines indicate either the single (5nM) or cumulative (25nM) SkQ1 dose administered to CLP mice in the main survival study (Experiment 1). The rat data serve as species comparison. Supplementary Fig. 4. Visualization of outcome for each individual CLP run. CLP was performed in six independent reiterations with 14-15 mice at each repetition (typically 5 mice/each group; the precise n indicated on each panel). Statistical assessment of outcome was performed o [file 6412682.f1.pdf]

Mouse Wellbeing and General Condition Score

			Mouse Number																															
		Observation	1st	2nd	3rd	4th	1st	2nd	3rd	4th	1st	2nd	3rd	4th	1st	2nd	3rd	4th	1st	2nd	3rd	4th	1st	2nd	3rd	4th	1st	2nd	3rd	4th	1st	2nd	3rd	4th
**Fur**	**0**	not alt.																																
	**1**	blunt/dirty																																
**Posture**	**0**	not alt.																																
	**1**	hunched																																
	**2**	flat and/or tremors																																
**Mobility**	**0**	not alt.																																
	**1**	slow/stiff movement when disturbed																																
	**2**	no movement when disturbed																																
**Alertness**	**0**	not alt.																																
	**1**	decreased																																
	**2**	no reaction																																
**Weight**	**0**	loss of 0.6g or more																																
	**1**	no loss to 0.5g loss																																
	**2**	any gain																																
**Startle reflex**	**0**	not alt.																																
	**1**	delayed																																
	**E**	none																																
		**Total score**																																

8 or more points = euthanasia

not alt. = not altered

time points of observation:	time points of observation:
1st	4th
2nd	5th
3rd	6th
