# Supplementary figures and images for: Mitochondria-Targeted Antioxidants SkQ1 and MitoTEMPO Failed to Exert a Long-Term Beneficial Effect in Murine Polymicrobial Sepsis
Source: Oxid Med Cell Longev. 2017 Sep 19;2017:6412682. doi: 10.1155/2017/6412682 (PMC5625755; doi:10.1155/2017/6412682)

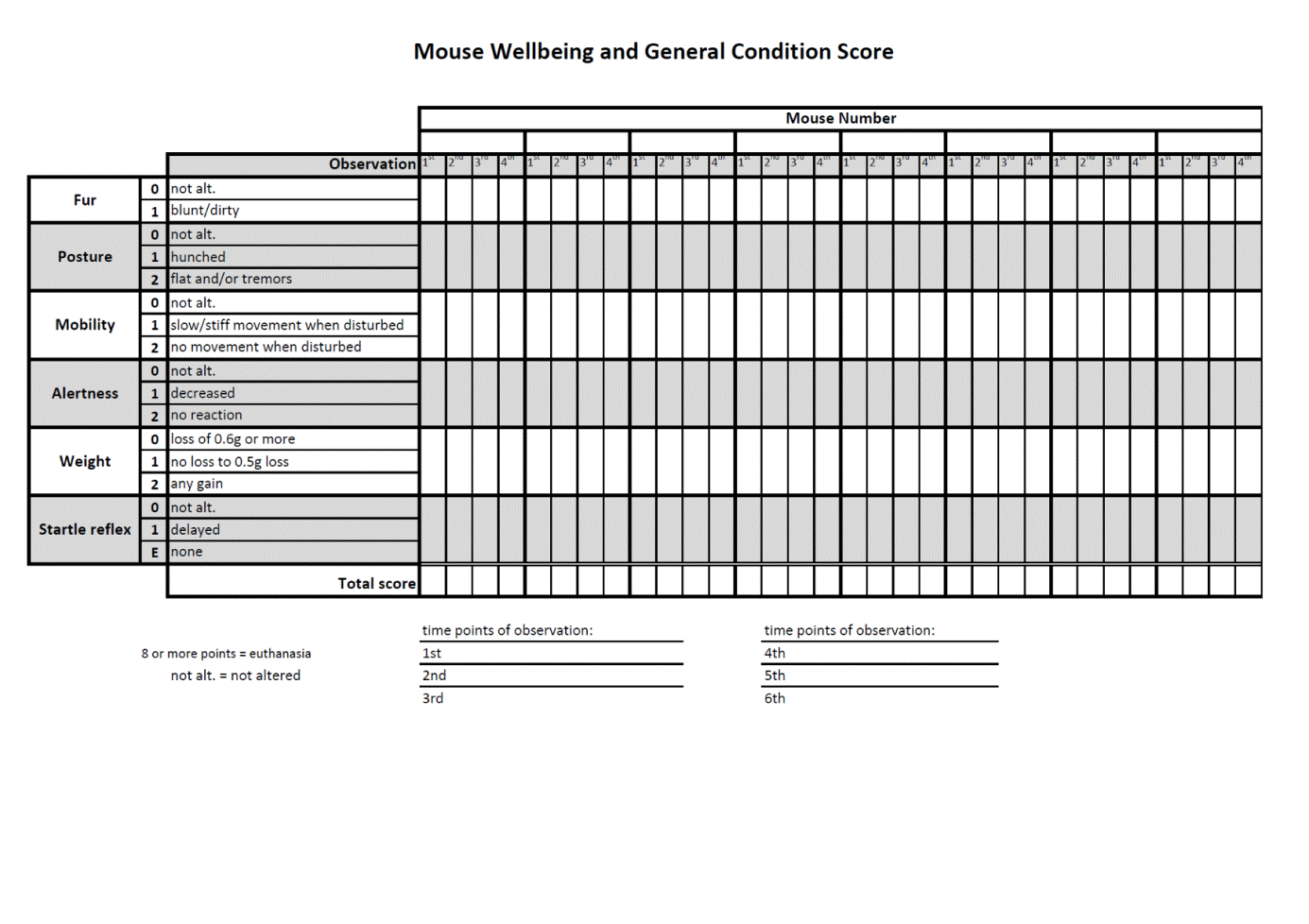

Supplement: Supplementary file 2 [file 6412682.f2.gif]

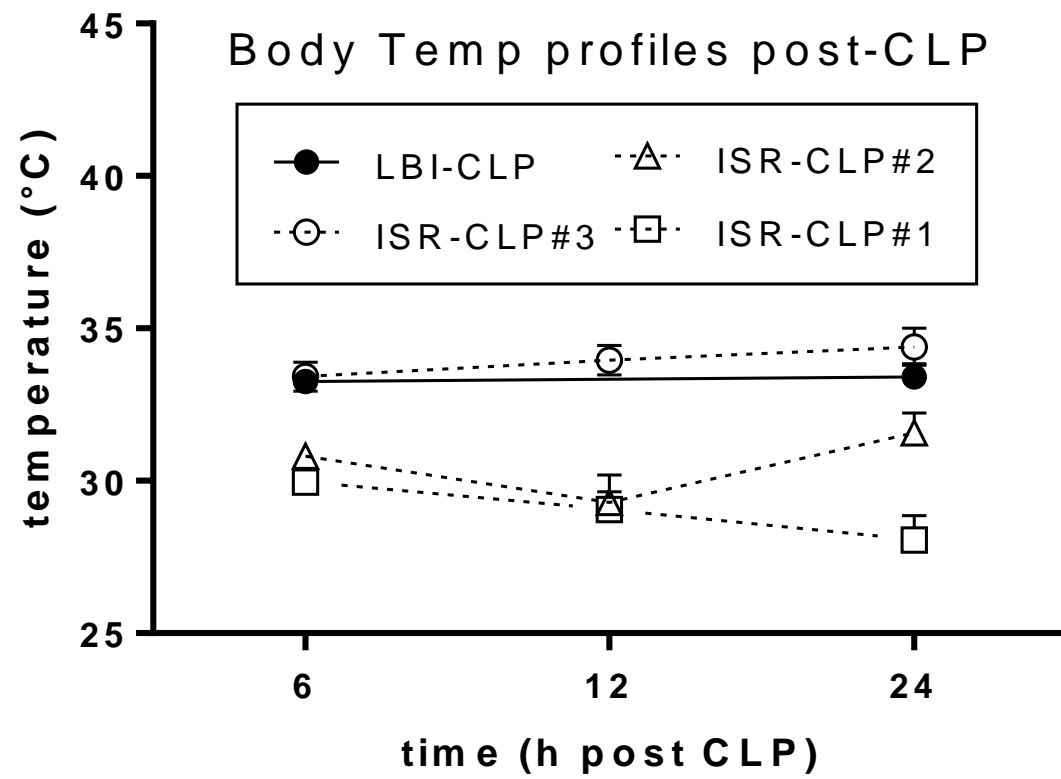

Supplement: Supplementary file 3 [file 6412682.f3.pdf]

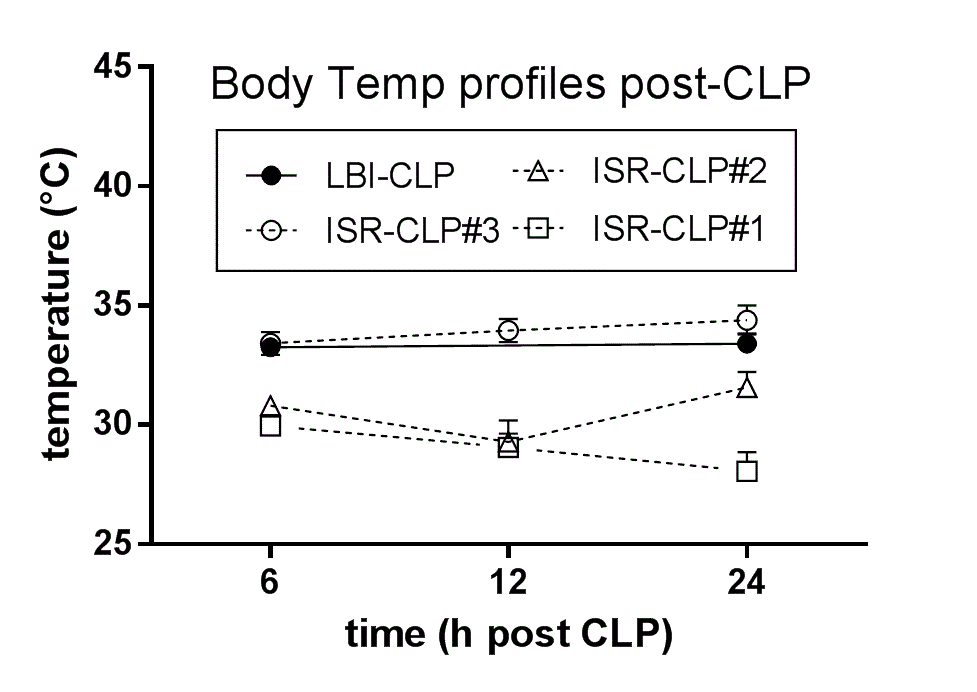

Supplement: Supplementary file 4 [file 6412682.f4.gif]

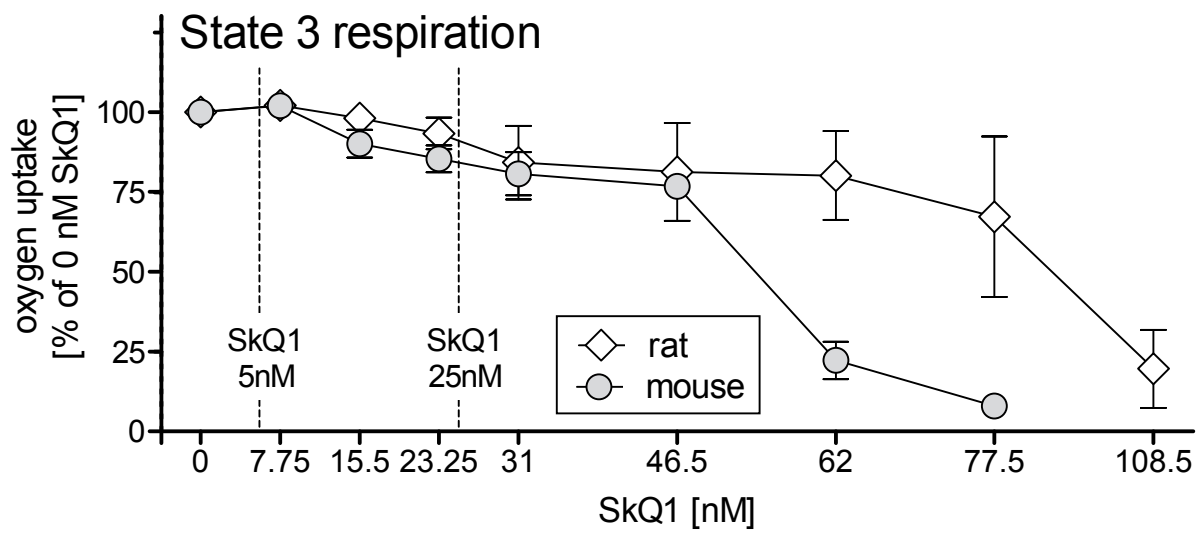

Supplement: Supplementary file 5 [file 6412682.f5.pdf]

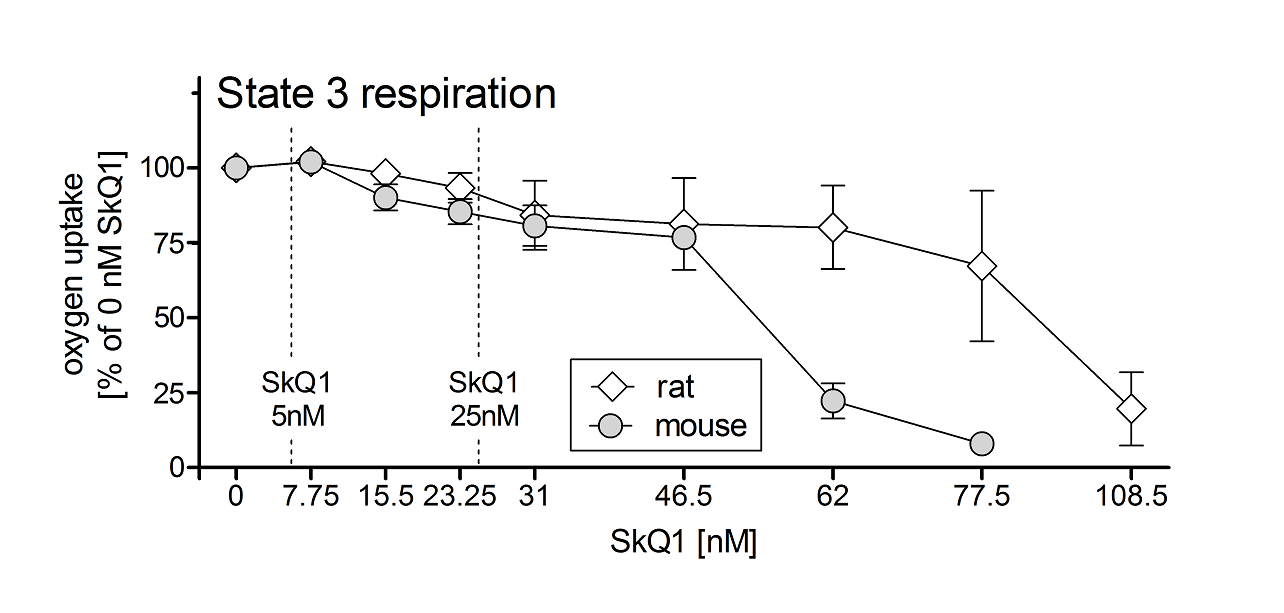

Supplement: Supplementary file 6 [file 6412682.f6.tif]

CLP Run 1

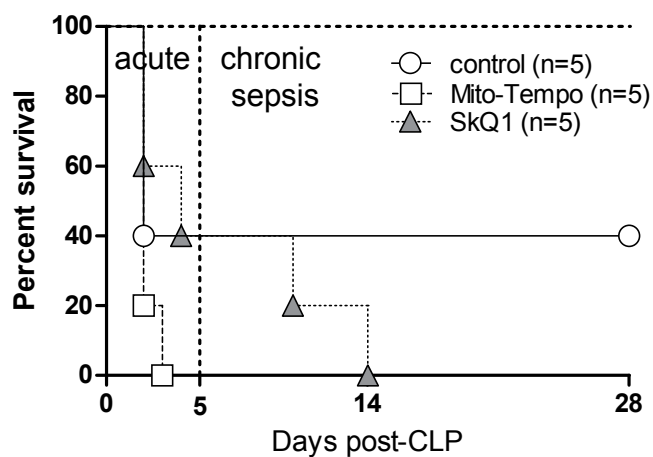

CLP Run 2

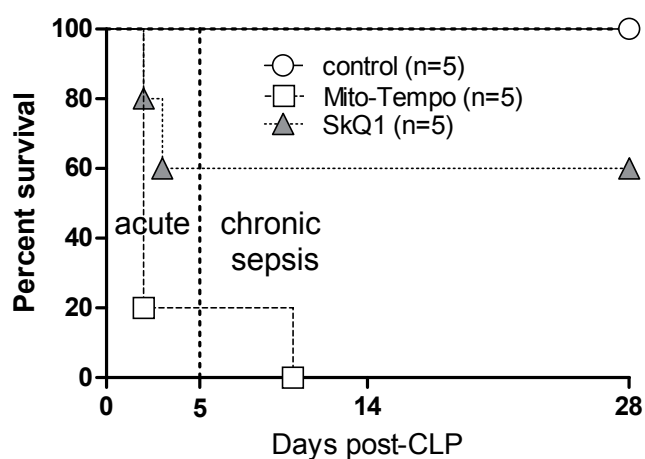

CLP Run 3

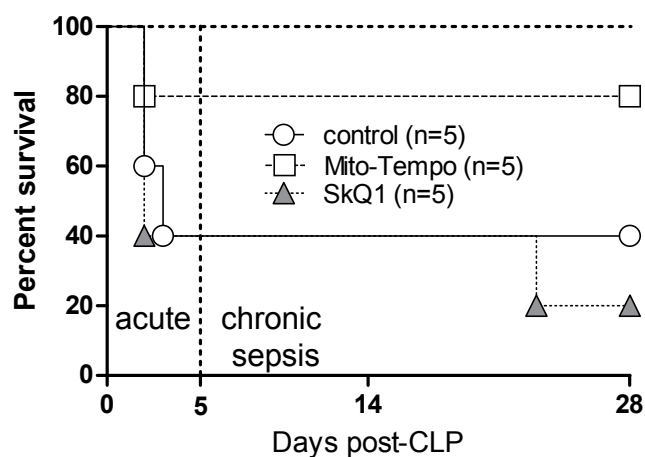

CLP Run 4

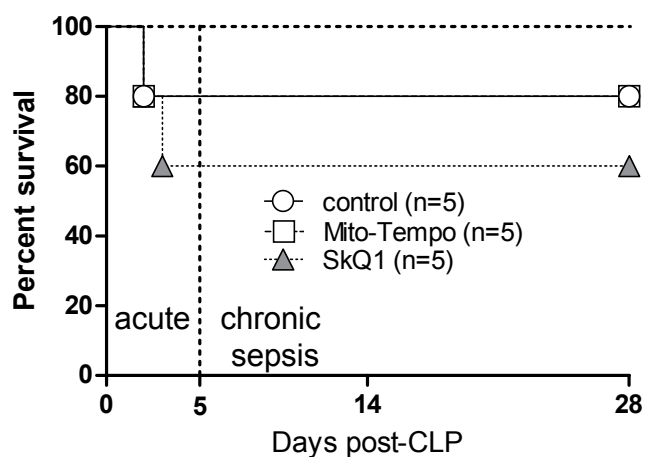

CLP Run 5

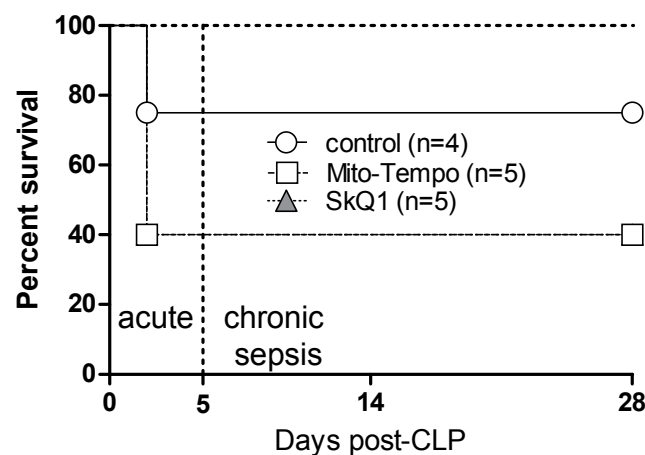

CLP Run 6

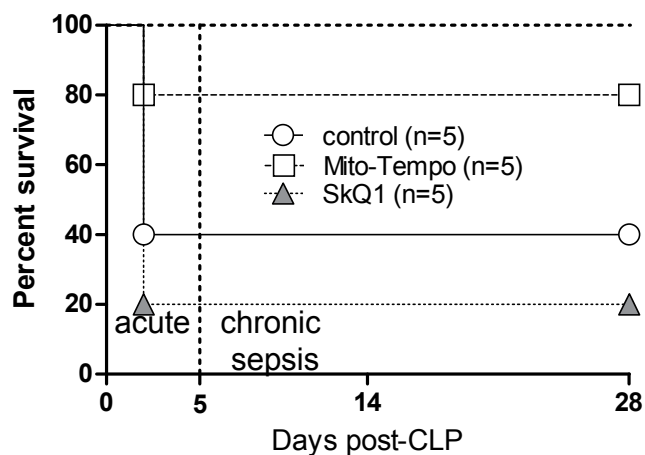

Supplement: Supplementary file 7 [file 6412682.f7.pdf]

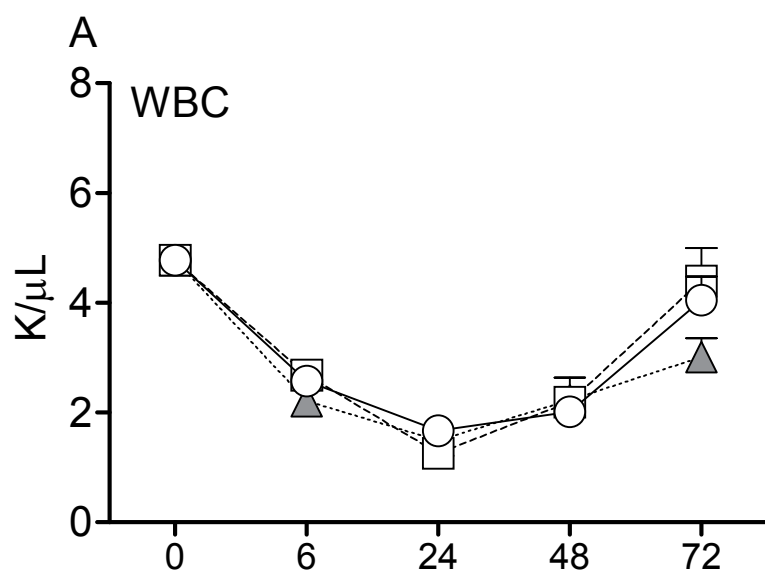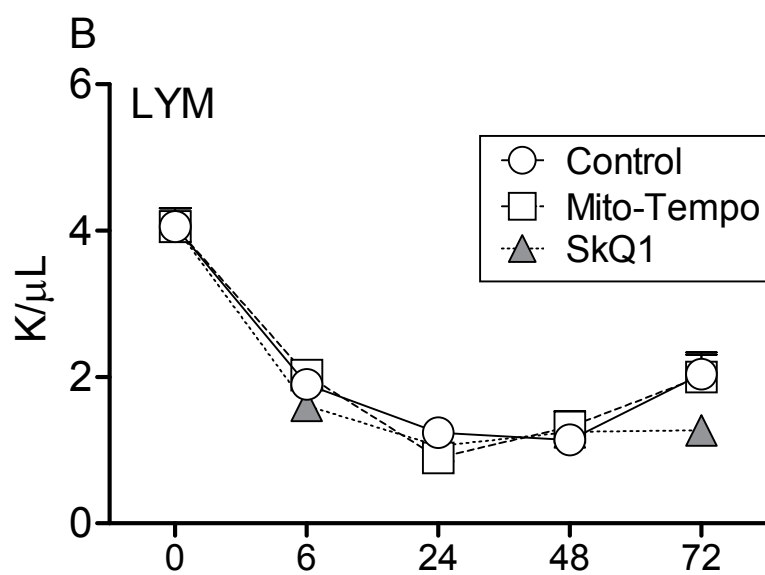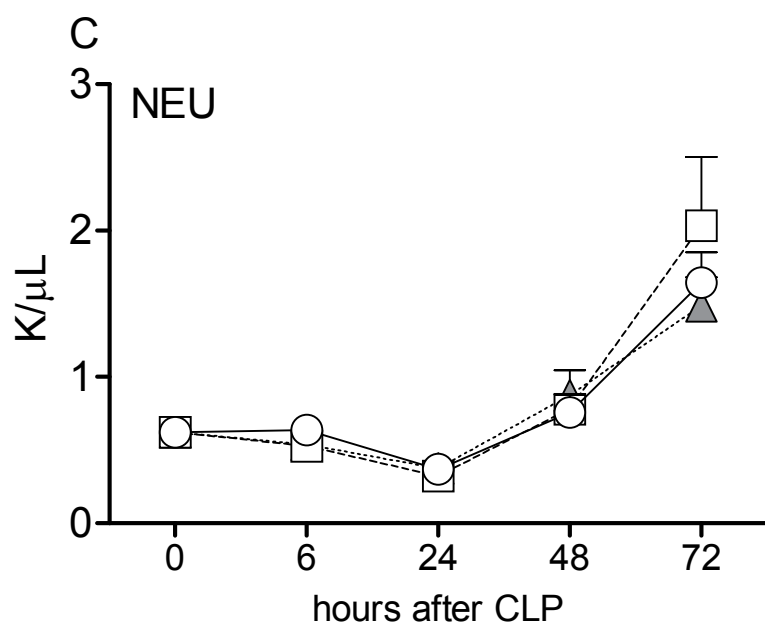

Supplement: Supplementary file 8 [file 6412682.f8.pdf]

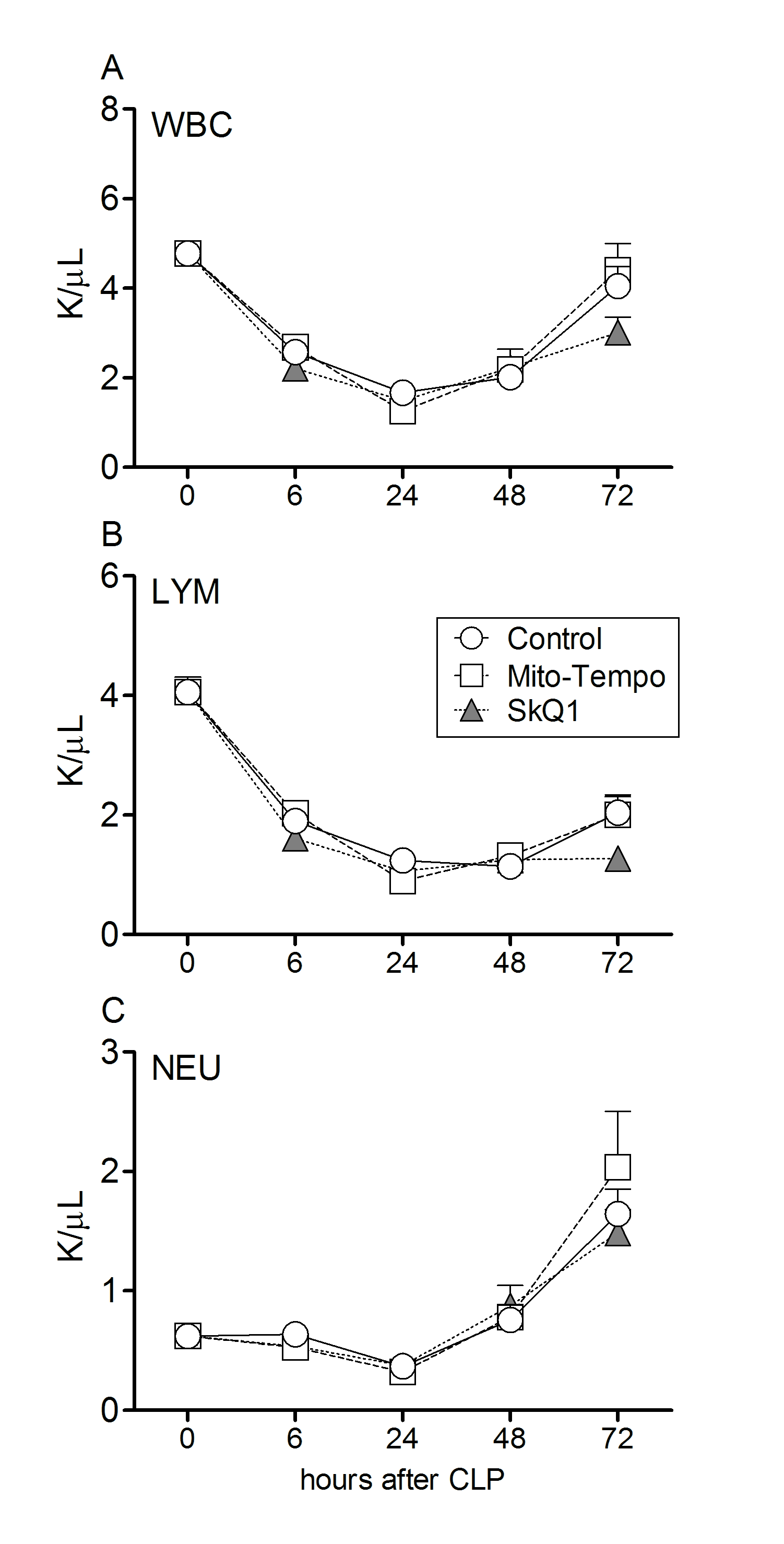

Supplement: Supplementary file 9 [file 6412682.f9.tif]

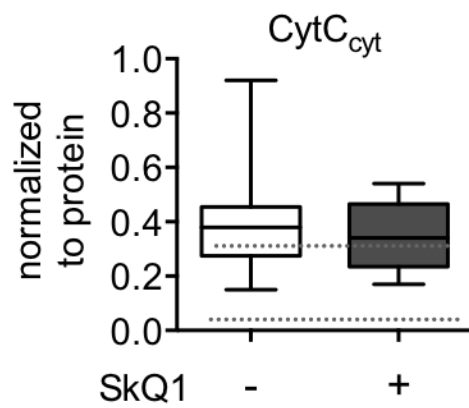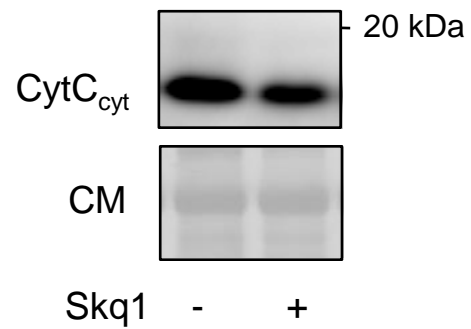

Supplement: Supplementary file 10 [file 6412682.f10.pdf]

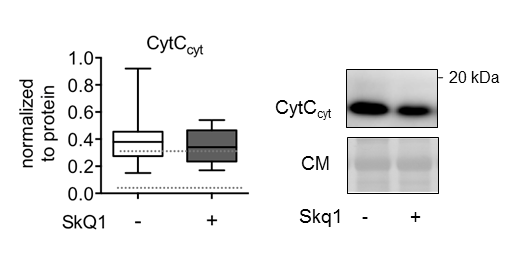

Supplement: Supplementary file 11 [file 6412682.f11.tif]

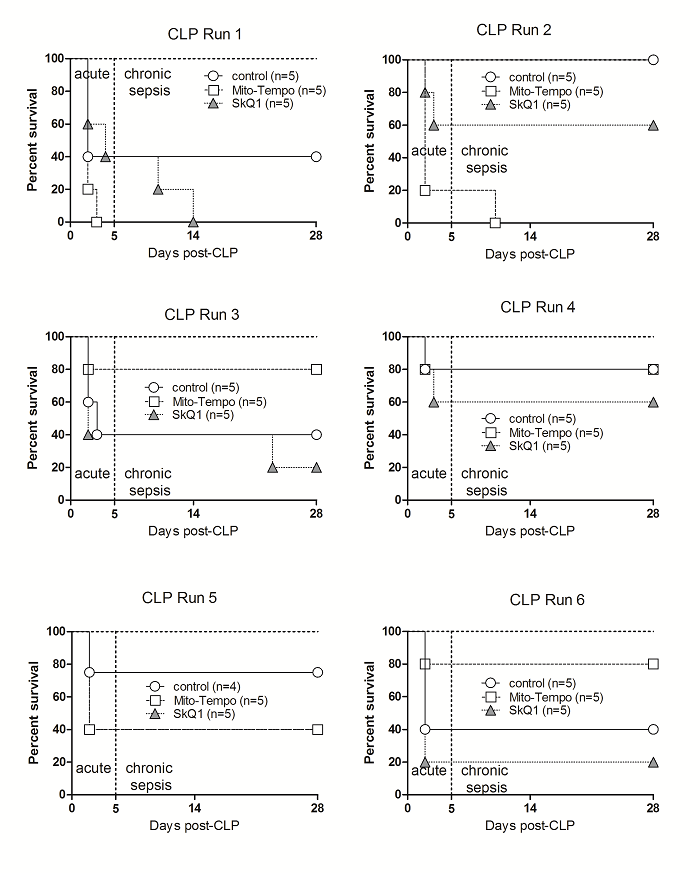

Supplement: Supplementary file 12 [file 6412682.f12.tif]
